# Implementation of infection control measures to prevent healthcare-associated transmission of severe acute respiratory coronavirus virus 2 (SARS-CoV-2)

**DOI:** 10.1017/ice.2020.1262

**Published:** 2020-10-12

**Authors:** Alexander J. Lepak, Daniel K. Shirley, Ashley Buys, Linda Stevens, Nasia Safdar

**Affiliations:** 1 Division of Infectious Diseases, Department of Medicine, University of Wisconsin School of Medicine and Public Health, Madison, Wisconsin; 2 Clinical Infection Control, UW Health University Hospital, Madison, Wisconsin; 3 Nursing Quality and Safety, UW Health University Hospital, Madison, Wisconsin; 4 William S. Middleton Memorial Veterans’ Affairs Medical Center, Madison, Wisconsin

The potential for nosocomial spread of severe acute respiratory coronavirus virus 2 (SARS-CoV-2) is a primary concern of public health experts, hospital epidemiologists, clinicians, healthcare institutions and patients, particularly because SARS-CoV in 2003 was associated with substantial nosocomial spread^1^ and SARS CoV-2 has a considerably high reproductive number.^2–4^ The reasons for efficient person-to-person transmission are multifactorial, including high-level viral shedding in the upper respiratory tract and documented presymptomatic, asymptomatic, and paucisymptomatic spread.^5–9^ In this study, we describe the infection control measures implemented and the relationship with SARS-CoV-2 test results in hospitalized patients.

The University of Wisconsin Health System (UW Health) includes 3 hospitals, with 672 beds and >120 clinics; it serves >600,000 patients in the Upper Midwest. The infection control program includes a special pathogens prevention multidisciplinary program that led the coronavirus disease 2019 (COVID-19) preparedness and response, including measures to prevent nosocomial transmission of SARS-CoV-2. The infection control measures instituted, time of implementation, and description of each intervention are listed in Table 1. Each intervention fell into 1 of 6 general categories: (1) personal protective equipment guidance and training, (2) testing guidance and algorithms, (3) monitoring of patients, visitors, and staff for signs and symptoms, (4) improving communication and patient care processes for patients with suspected or proven COVID-19, (5) implementation of electronic medical record decision support aids, and (6) control of physical environment with cohorting of suspected patients or patients and maintaining physical distancing.

**Table 1.** Implementation, Timing and Description of Infectious Control Measures Instituted in Response to the COVID-19 Pandemic

Date of Institution	Infection Control Measure	Description
Pre-existing	Staffing support	Clinical nurse specialist and care team leader budgeted to assist with COVID-19–confirmed patient when needed, putting less stress on staff
Pre-existing, 2003	Biocontainment unit	Unit wing designed with multiple airborne isolation rooms to allow for cohorted care of special pathogens; entire unit can be made to have negative air flow
Pre-existing, 2011	Clinical simulation center	PPE training in simulated situations
Pre-existing, 2014	Special pathogens team	Team included members from infectious diseases, safety, infection control, education, nursing, providers, and respiratory therapy. Quarterly team PPE and scenario training; regular meetings for preparedness planning
1/21/2020	PPE donning and doffing training for employees	Included one on one, on unit observations and training regarding COVID-19–specific PPE donning and doffing by infection control practitioners
1/21/2020	Special pathogen pager	Activated dedicated pager with on call infectious diseases physician to help with isolation, testing, and management questions
1/22/2020	Room entry log	Created and maintained log of persons entering the room of a confirmed or suspect COVID-19 inpatient (for contact-tracing purposes)
1/22/2020	Employee self-monitoring	Employees mandated to self-monitor for symptoms and report to EHS should they develop symptoms
1/23/2020	Special pathogens isolation sign updated	Required PPE for entry into room of patient with suspect or confirmed COVID-19: Respirator for ICU/IMC or aerosol generating procedures (barrier mask for general care COVID-19–positive patients), face shield, gloves, gown, extended use of PPE for 7 days of wear for N-95 and barrier mask
1/24/2020	Travel screening	Began screening all patients for pertinent travel history and symptoms upon entry into our system. EMR updates to assist with query and documentation of travel history
1/31/2020	Enhancements in the EMR	Screening questions developed for use in EMR with automatic electronic alerts to ensure the use of the correct type of isolation, patient placement and testing
2/14/2020	Biocontainment unit, ICU	Second biocontainment unit established for ICU care
2/21/2020	Implementation of the “COVID-19 huddle”	Immediate meeting with unit staff and provider to discuss plan for lab collection, patient placement, logistics, when an inpatient PUI is identified. Huddle included the following: REQUIRED staff for huddle: Charge RN or CTL (charge RN or CTL will lead huddle) MD/APP Primary RN or MA CONFIRM approval has been received from special pathogens MD that testing has been approved Designate staff that will enter room Only essential persons should enter using appropriate PPE No students, volunteers, interpreters should enter room Gather testing supplies (kit and PPE) Start log of individuals entering room – 2019 novel Coronavirus Post special pathogens sign on door and order isolation Collect tests as per lab specimen collection for 2019 novel coronavirus
3/2/2020	PPE donning and doffing observations on the COVID-19 units	Infection control team members performing on unit observation and feedback
3/5/2020	Student and volunteer restrictions	Cannot be involved with clinical evaluation or treatment of patients with acute respiratory illness; cannot enter rooms where PPE is required (eg, isolation)
3/9/2020	Travel guidelines for UW health employees	Cancel UW Health work travel outside Dane County; reconsider nonessential personal travel
3/11/2020	Change in visitor guidelines	All visitors screened for symptoms. 1 visitor/primary support per patient at one time. Exception: children’s hospital: 2 primary support per patient at one time; no siblings; no family members in OR suites. No limit to number of visitors at end-of-life
3/12/2020	Offering and promoting alternatives to in-person visits	Video visits with provider/consulting teams when possible. EVS and culinary staff refrain from room entry for patients with Special Pathogens isolation
3/12/2020	Unit-based training	COVID-19 traveling cart, unit based; implemented to supplement simulation center training Topics addressed: donning and doffing of PPE, updated patient isolation practices, low risk versus high risk scenarios (aerosol generating procedures), review NP swab collection, review where to find the COVID-19 resources
3/12/2020	PPE donning and doffing training in simulation center	Formal COVID-19–related PPE training sessions in the simulation center
3/13/2020	Employee testing site established	Ambulatory, off site, drive-through employee testing site implemented
3/13/2020	Increased EHS staffing & encouraging employee absenteeism	Guidance provided to employees: Self-monitor signs and symptoms twice daily Report any signs/symptoms to EHS COVID-19 testing, self-quarantine while awaiting results EHS will notify employee with results determine when it is possible to return to work
3/15/2020	Temporary work from home announced/rolled out	Staff may be eligible to work from home if their physical presence is not required to perform the essential functions of their role, as determined by department leadership
3/16/2020	RT-PCR testing in house	In house SARS-CoV-2 RT-PCR assays validated (NP swab) with significant improvement in capacity and turnaround time
3/17/2020	Designated entrance for patients/visitors and alternate entrance for employees	Physically separate and decrease close interactions between employees and visitors/public
3/18/2020	Special pathogens RNs as on-demand resource	Special pathogens RNs with prior intensive training were on call/available onsite 24/7 and served as content experts and on-site trainers as well as an “extra set of hands” for the complex care of these patients
3/18/2020	Ambulation for confirmed COVID-19 cases	Patients with confirmed COVID-19 can ambulate in room or on the COVID-19 unit (which is negative pressure, including hallway); must wear mask when outside of room
3/18/2020	Physical distancing update	Physical distancing posters rolled out. Directed at patients/visitors but a reminder for staff
3/18/2020	Special pathogens sign update	ICU/IMC/aerosol-generating procedures: gown, gloves, face shield, respirator, AII room required General care: gown, gloves, face shield, barrier mask
3/19/2020	COVID-19 patient transport update	Huddle to assess whether procedure can be done at bedside (eg, hemodialysis, x-rays, etc). Specific transport routes were predetermined and utilized. Patient and transport staff must wear PPE when outside room
3/20/2020	Universal masking and face shield	All personnel must wear barrier mask and face shield with any patient care contact
3/21/2020	Further visitor restrictions	No visitors, other than healthcare power of attorney Cafeteria closed to all patients/visitors
3/22/2020	Elective surgical procedures temporarily suspended	
3/22/2020	Activation of biocontainment units	The 2 biocontainment units were now dedicated to COVID-19–confirmed patients and PUIs only; improved processes to cohort staff as well
3/23/2020	Patient and visitor screening	All patients and visitors answered screening questions and had temperature recorded
3/23/2020	“Just in time” fit testing clinics	Ensured that healthcare providers who had not been fit tested and would need to wear a respirator were fit tested
3/28/2020	COVID testing for asymptomatic patients prior to certain procedures	
3/31/2020	Respiratory care unit in the ED	Designated respiratory care unit in ED to cohort patients with fever and respiratory symptoms Eliminated shared waiting rooms
4/13/2020	Daily inpatient symptom screening	Daily symptom screening of all hospitalized patients Documentation required in the EMR
4/21/2020	Admission testing	COVID-19 testing on admission for all inpatients
5/5/2020	Visitor restrictions modified	Only 1 visitor/primary support per patient per day, who must undergo screening prior to entry
6/15/2020	Physical distancing guidelines	Detailed guidelines regarding assessing spaces for maximum capacity, placing signage to ensure physical distance between people

Note. AII, airborne infection isolation; APP, advanced practice provider; CTL, care team leader; ED, emergency department; EHS, employee health services; EMR, electronic medical record; ICU, intensive care unit; IMC, intermediate care; MD, medical doctor; NP, nasopharyngeal; OR, operating room; PPE, personal protective equipment; RT-PCR, reverse-transcriptase polymerase chain reaction; PUI, person under investigation; RN, registered nurse.

As a measure of the success of these interventions, we examined the positivity rate for SARS-CoV-2 RT-PCR testing of inpatients from March 13, 2020, to June 25, 2020. All testing was performed using nasopharyngeal swabs with emergency-use authorization (EUA)-approved RT-PCR testing methods. Patients who were tested as outpatients, those tested in the emergency room or urgent care clinics, and those tested within the first 24 hours of an admission were excluded. Notably, repeated inpatient testing of individuals was, in general, directed toward those undergoing procedures, those in whom signs or symptoms suggested possible COVID-19, those with acute changes in status requiring intensive care unit (ICU) or intermediate (IMC) care, and/or based on provider judgment.

In total, 720 patients were tested >24 hours after admission to an inpatient unit, and the total number of inpatient SARS-CoV-2 tests was 1,007. The median age was 59 years (IQR, 40–69) and 52% were male. The reason for testing was skewed toward asymptomatic screening preceding procedures (71%). This finding was expected because repeat preprocedural testing was directed to be done within 48 hours prior to any aerosol-generating procedure. Of 1,007 inpatient tests, 59 tests (5.9%) were positive and 58 were known to be positive prior to inpatient testing (eg, positive prior to admission or as part of admission work-up). Thus, only 1 patient (0.1%) tested positive during an inpatient stay in which that patient was not known to have a history of a positive test. Over the study period, we had a sizeable COVID-19 inpatient population (112 inpatients with 1160 inpatient days) and a large at-risk pool of inpatients without COVID-19 (37,096 inpatient days).

For the single positive inpatient without a prior history of SARS-CoV-2, chart review revealed that this adult patient lived in a community setting, had mild symptoms (sinus congestion, eye pain, and cough) that started 10 days prior to admission, and was self-isolating at home. The patient presented with a myocardial infarction before universal admission testing was instituted, and the prior mild respiratory symptoms were not noted. On hospital day 4, the patient tested positive as part of pre-procedure screening. We believe that infection was present from community exposure prior to admission; therefore, we did not find any laboratory-confirmed cases suggestive of possible nosocomially acquired SARS-CoV-2 infection despite a substantial inpatient population with and without COVID-19. It has been suggested that false-negative results may occur, but negative-to-positive conversion has rarely occurred at our institution (<1%).^10^ Importantly, we were able to achieve these results without routine, serial testing of asymptomatic healthcare workers (HCWs), and we had a low threshold for testing HCWs with symptoms with a 1% rate of infection in our HCWs.

Our study has several limitations. First, this was a retrospective observational study. Second, because testing was limited to inpatient setting, we were not able to ascertain symptom onset after discharge, which may have resulted in testing elsewhere. However, we examined all positive ambulatory tests and did not find any positive results in patients within 7 days of discharge from our hospital. Finally, we were unable to examine the relative effect of each individual infection control measure.

Our study has a number of strengths. As the single positive case we found demonstrates, it can be difficult to identify all potential positive patients by history taking alone. Thus, we strongly believe that universal testing of patients admitted to the hospital should be performed. This testing should be followed by targeted testing based on daily, protocol-driven screening questions to determine whether any symptoms have changed that suggest possible COVID-19. These first 2 measures aim to rapidly identify patients that should be placed in transmission-based isolation and to help prevent inadvertent spread. However, additional measures are obviously necessary to prevent nosocomial spread from known SARS-CoV-2–positive patients who may need complex medical care including intensive care, multiple-specialty care, invasive procedures or surgery, and intrahospital transport. These measures include meticulous infection control measures described here. In conclusion, using iterative implementation of infection control measures we were able to care for numerous COVID-19–infected and –uninfected patients without any cases of nosocomial spread.
